# Abdominal Aortic Aneurysm Morphology as an Essential Criterion for Stratifying the Risk of Aneurysm Rupture

**DOI:** 10.3390/jcm11040933

**Published:** 2022-02-11

**Authors:** Natalia Niklas, Piotr Gutowski, Arkadiusz Kazimierczak, Paweł Rynio

**Affiliations:** Department of Vascular Surgery, Pomeranian Medical University in Szczecin, Al. Powstańców Wielkopolskich 72, 70-111 Szczecin, Poland; nniklas488@gmail.com (N.N.); piotr_gutowski@poczta.onet.pl (P.G.); biker2000@wp.pl (A.K.)

**Keywords:** abdominal aortic aneurysm, aneurysm risk rupture, aneurysm wall structure

## Abstract

The current stratification model of aneurysm rupture seems to be insufficient in some clinical cases. In our study, we determined the differences in wall structure between ruptured and unruptured aneurysms. We obtained computed tomography angiograms and categorized them into the following three groups, consisting of 49 patients each: the group with ruptured abdominal aortic aneurysms (rAAA), symptomatic (sAAA), and asymptomatic (aAAA). The three-dimensional AAA anatomy was digitally reconstructed for each patient through semi-automatically obtained segmentation, and each aneurysm was distinguished by the following three parameters: AFL (aneurysm flow lumen), ILT (intraluminal thrombus), and calcifications. The AFL volume was greater in rAAA compared with aAAA (*p* = 0.004), the ILT volume was greater in aAAA than in rAAA (*p* = 0.013), and the AFL/ILT surface ratio was bigger in rAAA than in aAAA (*p* < 0.001), sAAA than in aAAA (*p* = 0.033), and rAAA than in sAAA (*p* = 0.016). AFL/ILT surface*100 was defined as an independent predictive factor of rAAA to aAAA (OR 1.187; 95% CI 1.099–1.281), to sAAA (OR 1.045; 95% CI 1.004–1.087), and in sAAA vs. aAAA (OR 1.067; 95% CI 1.017–1.119). Consequently, the wall of rAAA differs significantly from unruptured aneurysms. The AFL/ILT surface ratio might indicate an increased risk of aneurysm rupture and the occurrence of symptoms in AAA.

## 1. Introduction

Abdominal aortic aneurysm (AAA) represents a significant public health problem. It is a typical disease of elderly males, and it accounts for 1% of the deaths in men over 65 years old and more than 175,000 deaths worldwide [1]. The mortality rate associated with the rupture of abdominal aortic aneurysm is very high, up to 80% [1], highlighting the need for non-invasive predictors of aneurysm rupture risk. 

The current stratification model of aneurysm rupture risk is mainly based on the diameter of the aneurysm, with the threshold for considering abdominal aortic aneurysm repair being ≥5.5 cm in men. The risk of rupture in women, especially for small-sized AAAs, is about four times higher than in men [2]. Moreover, they have slightly higher perioperative mortality after open surgery on a ruptured aneurysm. However, this is probably due to the older age upon admission [3]. Consequently, the acceptable threshold of aneurysm diameter for surgical repair in women is ≥5 cm. The rapid growth rate of abdominal aortic aneurysms (≥1 cm/year) should indicate that a prompt consultation with a vascular surgeon [4] is necessary. However, every case of AAA elective repair should be considered individually [5]. Recent studies show that up to one in five small AAAs (≤5 cm) do rupture [6], pointing out the need to expand research in this field. Stratification of rupture risk in patients remaining under surveillance is still demanding for healthcare professionals.

AAA rupture occurs when the wall stress exceeds the strength of the aortic wall. The intraluminal thrombus (ILT), practically found in every aneurysm of clinically meaningful size, is suggested to have a protective role against aneurysm rupture, by diminishing the tension on the wall [7] and the peak wall stress (PWS) [8]. Other research associated the ILT thickness with optimal nidus for creating an inflammatory environment, apoptosis of vascular smooth muscle cells, and elastin degradation [9]. 

It is generally assumed that calcifications can affect the strength of soft tissue and, therefore, significantly impact aneurysm rupture [10]. However, recently, not much research has been conducted in this field on patients matched for aortic diameter and modern techniques. So far, a positive correlation has been found between the lumen area in ruptured aneurysms and the lumen area in asymptomatic individuals [11], which encourages further investigations of the potential clinical applications of these parameters.

The currently used scheme for estimating the risk of aneurysm rupture could be enriched by more details of the aneurysm wall structure itself, thus completing the model, based mainly on the diameter of the aneurysm, which, in some clinical cases, is insufficient. A combination of modern diagnostics methods with the principal involvement of three-dimensional computed tomography scans and the segmentation of specific pathology would allow us to obtain a patient-specific prediction model for aneurysm rupture.

Therefore, in this study, we sought to explore the differences in wall structure between ruptured and unruptured aneurysms (divided into symptomatic and asymptomatic), such as the surfaces and volumes of ILT (intraluminal thrombus), calcifications, and AFL (aneurysm flow lumen). 

## 2. Materials and Methods

Computed tomography angiograms (CTA) were collected from patients diagnosed with AAA from January 2010 to December 2018 from the central patient database at the Department of Vascular Surgery at our center. Three groups were distinguished, including patients with ruptured abdominal aortic aneurysms (rAAA), asymptomatic aneurysms (aAAA), and symptomatic aneurysms (sAAA). 

A symptomatic patient was defined as a person with a documented diagnosis of an abdominal aortic aneurysm, with clinically distinct symptoms related to the aneurysm, after excluding other possible causes, and with precise annotations in the hospital medical records. An asymptomatic aneurysm was defined as an aneurysm without clinical symptoms discovered fortuitously during radiological examinations. CTA of the patients with ruptured AAA was obtained after their arrival at the hospital in a life-threatening state. Patients from all groups underwent surgical treatment afterwards. Patients’ baseline characteristics are presented in Table 1.

All subjects had isolated abdominal aortic aneurysms with aortic diameters >5.5 cm in men and >5.2 cm in women, arising from the management of abdominal aortic aneurysms of that time [12]. Patients with a thoracoabdominal aneurysm, or with previously implanted abdominal stent grafts, were excluded from the research. 

### 2.1. Image Analysis

**Figure 1:**
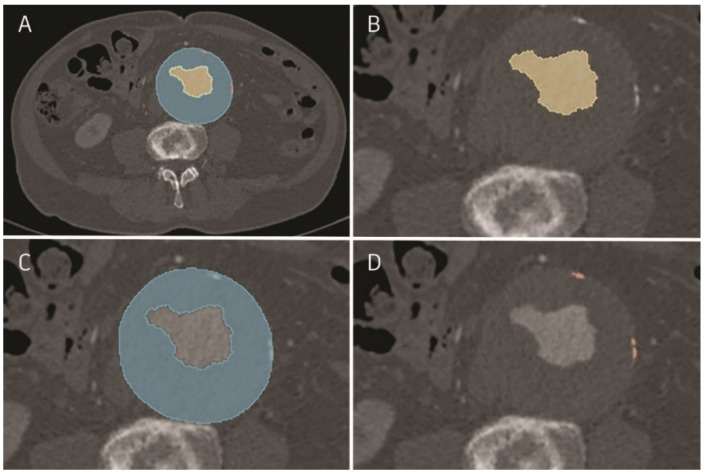
Semi-automatically obtained segmentation made in *3dSlicer* program: (**A**) computed tomography angiogram with segmented AFL, ILT, and calcifications; (**B**) aneurysm flow lumen (AFL) in AAA; (**C**) intraluminal thrombus (ILT) in AAA. Obtained region of ILT could contain calcifications; (**D**) calcifications in AAA.

Image analysis was performed on preoperative CTA obtained from the patients. Three-dimensional AAA anatomy was digitally reconstructed from CTA for each patient through semi-automatically obtained segmentation using open-source software *3dSlicer* 4.11.0 (http://www.slicer.org, accessed on 30 September 2020). In each CTA, infrarenal abdominal aortic aneurysm was segmented up to the aortic bifurcation into the common iliac arteries. Moreover, each aneurysm was distinguished according to the following 3 leading parameters: AFL (aneurysm flow lumen), ILT (intraluminal thrombus), and calcifications (Figure 1A). Each of the analyzed parameters was considered in terms of volume (cm^3^) and surface (mm^2^).

AFL was differentiated by a plug-in “Grow from seeds” to achieve complete segmentation by drawing segments inside a certain structure. This method is based on the master volume that uses the intensity of the segmented book, so the final segment boundaries were placed where the master volume changes abruptly between aneurysm flow lumen and intraluminal thrombus (Figure 1B). By subtracting AFL from the total AAA area, the ILT was obtained (Figure 1C). Finally, the calcifications were derived by filling the precise segment based on the volume intensity range, with the registration of attenuation threshold values expressed in Hounsfield units (HU) (Figure 1D). The segmentation was eventually verified by an experienced vascular surgeon and manually adjusted if necessary.

### 2.2. Statistical Analysis

Statistics were performed using Statistica 13.0 software (TIBCO Software, Palo Alto, CA, USA). The asymptomatic and symptomatic groups were matched to the ruptured aneurysm group using propensity score matching. The factors used in generating propensity scores were the patient’s gender and aortic diameter. The maximal aneurysm diameter was employed for logistic regression, then to determine propensity. As a result, we obtained three groups consisting of 49 patients each. The hypothesis of normality of distribution was tested with Shapiro–Wilk and Lilliefors tests. The standard deviation and interquartile range were presented with mean values, when appropriate. All comparisons between continuous variables were performed with the Kruskal–Wallis test. The nominal variables were compared using the Pearson chi-square test. The Kruskal–Wallis test, or the Tukey test, was used for post hoc analysis. The main variables were correlated with Spearman’s rank test. A *p*-value <0.05 was considered statistically significant. Based on the analysis of ROC curves, cut-off points for the continuous variables were determined for the variables with a statistically significant AUC. Subsequently, the cut-off points were used to calculate the odds ratio for ruptured, asymptomatic and symptomatic aneurysms.

## 3. Results

Ruptured abdominal aortic aneurysms have a greater AFL volume (*p* = 0.004) and AFL surface (*p* = 0.004) compared with aAAA. Asymptomatic abdominal aortic aneurysms have a greater ILT volume compared with rAAA (*p* = 0.013). However, no statistically significant relationship was found regarding the ILT surface. The calcification volumes are greater in aAAA than in rAAA (*p* < 0.001), but also in sAAA than in rAAA (*p* < 0.001). A similar observation was made regarding its surface, which is greater in aAAA than in rAAA (*p* < 0.001), as well as in sAAA than in rAAA (*p* < 0.001). The AFL/ILT volume ratio is bigger in rAAA compared with aAAA (*p* < 0.001), and the same is true for the AFL/ILT surface ratio (*p* < 0.001). Symptomatic abdominal aortic aneurysms have a greater AFL/ILT volume ratio compared with aAAA (*p* = 0.011), as well as a greater AFL/ILT surface ratio (*p* = 0.033). Moreover, in rAAA vs. sAAA, a greater AFL/ILT surface ratio (*p* = 0.016) was observed (Table 2). The total volume (cm^3^) and maximal aneurysm diameter (mm) (Table 2) did not differ between particular groups.

An AFL volume > 181.56 cm^3^ (OR 7.170; 95% CI 2.428–21.174; *p* < 0.001), AFL surface > 20,492.20 mm^2^ (OR 5.953; 95% CI 2.410–14.701; *p* < 0.001), ILT volume < 172.16 cm^3^ (OR 4.786; 95% CI 1.957–11.704; *p* < 0.001), AFL/ILT volume ratio > 0.43 cm^3^ (OR 11.316; 95% CI 4.042–31.360; *p* < 0.001), and AFL/ILT surface*100 ratio >0.40 mm^2^ (OR 13.895; 95% CI 4.676–41.285; *p* < 0.001) were identified as independent predictive factors for aneurysm rupture risk when compared to the group of asymptomatic aneurysms (Table 3). A calcification surface < 782.12 mm^2^ (OR 8.366; 95% CI 3.294–21.249; *p* < 0.001) and calcification volume < 1.35 cm^3^ (OR 8.044; 95% CI 3.218–20.105; *p* < 0.001) seem to characterize a ruptured aneurysm. However, there is no linear correlation.

The predictive factors for aneurysm rupture compared to symptomatic aneurysms are as follows: AFL volume > 189.26 cm^3^ (OR 2.591; 95% CI 1.077–6.232; *p* = 0.034), AFL surface >18,808.70 mm^2^ (OR 3.474; 95% CI 1.502–8.033; *p* = 0.004), and AFL/ILT surface*100 ratio > 0.45 mm^2^ (OR 2.991; 95% CI 1.310–6.826; *p* < 0.001) (Table 4). The analysis of the ROC curves demonstrated that a calcification surface < 1090.98 mm^2^ (OR 7.153; 95% CI 2.874–17.801; *p* < 0.001), calcification volume < 1.57 cm^3^ (OR 6.250; 95% CI 2.353–16.598; *p* < 0.001), and calcification/ILT volume*100 ratio < 0.01 cm^3^ (OR 2.450; 95% CI 1.050–5.713; *p* = 0.038) characterize ruptured aneurysms.

However, for the occurrence of symptomatic abdominal aneurysms plays an important role: ILT volume <170.12 cm^3^ (OR 3.450; 95% CI 1.413–8.426; *p* = 0.007), AFL/ILT volume*100 ratio >0.47 cm^3^ (OR 3.552; 95% CI 1.544–8.170; *p* = 0.003), and AFL/ILT surface*100 ratio >0.41 mm^2^ (OR 2.966; 95% CI 1.305–6.744; *p* = 0.01) (Table 5). 

It was determined that Spearman rank correlation would be used to measure the degree of association between the two variables. A solid positive relationship was found between the total volume and ILT surface (0.91), calcification volume and calcification surface (0.95), AFL volume and AFL surface (0.94), and AFL/ILT volume and surface ratio (0.87). Moreover, a strong positive relationship was found between the AFL surface and ILT surface (0.82), calcifications volume and calcifications/ILT volume ratio (0.82), calcifications surface and calcifications/ILT volume ratio (0.79), total volume and ILT volume (0.75), AFL volume and ILT surface (0.71), and maximum diameter and total volume (0.70). A strong negative relationship was found between the ILT volume and AFL/ILT volume ratio (−0.73). 

## 4. Discussion

Abdominal aortic aneurysms constitute a significant public health problem, as they can lead to life-threatening complications. However, the currently functioning model of aneurysm rupture risk stratification seems to be incomplete and should be enriched with the individual features of the aneurysm wall structure.

The role of ILT in aneurysm rupture risk is controversial, and is still a subject of research. This study suggests that the ILT volume is greater in aAAA compared to rAAA.

No relationship between ILT volume in rAAA and intact AAA was found in the study from 2014, which used CTA to compare the ILT volume in a smaller group of 28 patients with ruptured aneurysms, and 56 patients with intact (asymptomatic) aneurysms [13]. However, the ILT volume was calculated using a previously validated semi-automated workstation protocol [14], which differs from the methodology used for ILT determination in this study; additionally, the small sample size could affect the results of the research. 

Filinger et al. [15] claimed that thrombus characteristics, such as the maximum thrombus thickness and circumference, were not significantly different in a sample of 100 ruptured and 100 intact abdominal aortic aneurysms. However, this study only used conventional 2D CT axial sections and simplified methods of measurements, without evaluation of 3D reconstructions.

Hans et al. [16] analyzed the ILT volume on CT scans obtained from 67 patients with unruptured AAAs and from31 patients with ruptured AAAs, and recorded its morphology using AutoCAD 2000 software. The researchers reported that ruptured AAAs had a significantly greater ILT volume when compared with intact AAAs. However, obtaining the total volume of ILT via the sum of the volume of each disc in AAAs, while most abdominal aortic aneurysms are marked by irregular lumens and asymmetric structures, could introduce bias. A modern approach, based on the detailed segmentation of the intraluminal thrombus, enables the accurate determination of ILT volume and surface, considering the individual features of every aneurysm. Moreover, the investigators suggested that the ILT-to-aneurysm ratio is irrelevant in predicting rupture risk. In our study, the calculated ratios, particularly the AFL/ILT surface, seem to be the most important criterion for determining the risk of aneurysm rupture and the occurrence of symptomatic aneurysms. 

A recent meta-analysis of eight studies, investigating the role of intraluminal thrombus volume in aneurysm rupture risk [17], found a greater ILT volume in those aneurysms that had ruptured compared with intact AAAs. However, a significantly larger diameter was observed in ruptured aneurysms than in asymptomatic intact AAAs. Moreover, only three studies matched for diameter, and their further subanalysis revealed no significant differences between groups, concerning ILT volume. Other prospective studies are needed to clarify the practicality of obtaining thrombus volume measurements in patients remaining under surveillance. Our research may suggest a crucial role of ILT volume in aneurysm rupture.

Despite much scientific research, there is no clear relationship between the occurrence of calcification and the development of AAA, and whether the connection is direct or a result of common risk factors. Notwithstanding, it is generally considered that there is a positive correlation between AAA calcification and rupture risk. Buijs et al. [18] performed a semi-quantitative analysis of AAA calcification using the AAC-8 score [19]. The investigators reported the highest AAC-8 score for symptomatic abdominal aortic aneurysms (sAAA), then for ruptured abdominal aortic aneurysms (rAAA), and patients with elective AAA had the lowest AAC-8 score. Thereupon, this study shows a clear association between aortic calcification and aneurysm rupture risk, thereby indicating that the degree of aortic wall calcification correlates with the development of symptoms in patients with AAA. Nevertheless, this study has limitations, as the AAC-8 scale is mainly observatory dependent, and the results may primarily depend on the experience of a physician assessing the computer tomographs. An automated method for evaluating the calcium burden in aortic walls would unify the rating system and be a more objective tool. Interestingly, ruptured aneurysms had the lowest calcification rates in our research, and the diverse methodology used could significantly impact the results. Undeniably, additional research is needed to clarify the degree of calcification and the occurrence of symptoms in patients with AAA.

There are not many studies assessing the significance of lumen area in intact and ruptured aneurysms. Siika et al. [11] reported that the diameter, cross-sectional lumen area, and the cross-sectional total vessel area were larger in ruptured abdominal aneurysms compared with aAAA. Moreover, no differences were found in the ILT area between ruptured and asymptomatic AAA, which strictly correlates with the results of our study. The authors tried to find a biochemical explanation for this phenomenon by analyzing the peak wall stress (PWS) and peak wall rupture index (PWRI), which were not the subject of our study. However, the lumen area was positively correlated with both parameters, suggesting that an increased lumen area is associated with higher biochemical stress. Moreover, based on our results, lumen area measurements could be used as a possible predictor for risk of aneurysm rupture.

A comprehensive approach to the topic, with a three-dimensional analysis of the abdominal aortic aneurysm wall structure, and a combination of all three parameters (AFL, ILT, and calcifications), on a relatively large group of patients, are features that indicate the innovation of our research. Moreover, compared to the other studies mentioned above, the groups of aAAA and sAAA patients were matched with the group of patients with rAAA in maximal aneurysm diameter.

Correlations between specific parameters could be implemented in clinical practice by selecting a smaller number of factors, useful for individualized measurements of the aneurysm rupture risk.

## 5. Conclusions

The structure of rAAA differs significantly from an unruptured aneurysm. Those differences might contribute to the stratification of the risk of aneurysm rupture. Smaller AFL and AFL/ILT surface and volume ratios, and a higher ILT volume, seem to be protective against aneurysm rupture. A calcification surface < 782.12 mm^2^ (OR 8.366; 95% CI 3.294–21.249; *p* < 0.001) and calcification volume < 1.35 cm^3^ (OR 8.044; 95% CI 3.218–20.105; *p* < 0.001) seem to characterize ruptured aneurysms. A smaller ILT volume and higher AFL/ILT volume and surface ratio were identified as predictive factors for the occurrence of symptoms in asymptomatic aneurysms. Moreover, the AFL/ILT surface may correlate with an increased risk of aneurysm rupture and the occurrence of symptoms in AAA.

## Figures and Tables

**Table 1:** Patients’ baseline characteristics. HTN, hypertension; CAD, coronary artery disease; CABG, coronary artery bypass grafting; HF, heart failure; DM, diabetes mellitus; CKD, chronic kidney disease; PAD, peripheral artery disease; COPD, chronic obstructive pulmonary disease. Continuous variables are presented as mean ± standard deviation (SD), and categorical variables are presented as number (%).

Variable	Asymptomatic (*n* and % or ±SD)	Symptomatic (*n* and % or ±SD)	Ruptured (*n* and % or ±SD)	*p* Value
Age, years	71.57 (±8.82)	72.61 (±8.61)	76.35 (±10.48)	0.032
Age > 80	12 (24.49%)	10 (20.41%)	22 (44.90%)	0.018
Male gender	47 (95.92%)	37 (75.51%)	38 (77.55%)	0.013
HTN	34 (69.39%)	37 (75.51%)	29 (59.18%)	>0.1
CAD	21 (42.86%)	15 (30.61%)	11 (22.45%)	>0.1
CABG	6 (12.24%)	5 (10.20%)	2 (4.08%)	>0.1
Arrhytmia	8 (16.33%)	10 (20.41%)	8 (16.33%)	>0.1
HF	13 (26.53%)	3 (6.12%)	6 (12.24%)	0.015
DM	8 (16.33%)	7 (14.29%)	7 (14.29%)	>0.1
CKD	5 (10.20%)	2 (4.08%)	6 (12.24%)	>0.1
Stroke	6 (12.24%)	6 (12.24%)	4 (8.16%)	>0.1
PAD	9 (18.37%)	10 (20.41%)	5 (10.20%)	>0.1
COPD	10 (20.41%)	5 (10.20%)	7 (14.29%)	>0.1
Obesity	6 (12.24%)	8 (16.33%)	5 (10.20%)	>0.1

**Table 2:** The values for particular parameters for the following three groups: asymptomatic, symptomatic and ruptured aneurysms. *p* values are shown for all three groups and *p* values for post hoc Kruskal–Wallis test between particular groups are mentioned. Moreover, the comparison of total volume and maximal diameter obtained in asymptomatic, symptomatic, and ruptured abdominal aortic aneurysms was shown. There are no significant differences in the Kruskal–Wallis test, indicating that the aneurysm volume and diameter did not differ between groups.

	aAAA	sAAA	rAAA	*p*-Value *	*p*-Value Post Hoc rAAA vs. aAAA	*p*-Value Post Hoc aAAA vs. sAAA	*p*-Value Post Hoc rAAA vs. sAAA
Maximal aneurysm diameter [mm]	82.52 (IQR 13.10)	82.18 (IQR 14.00)	83.65 (SD 14.00)	>0.1			
Total aneurysm volume [cm^3^]	378.21 (IQR 147.16)	344.07 (SD 144.66)	381.59 (IQR 187.14)	>0.1			
AFL volume [cm^3^]	116.83 (69.97)	143.76 (101.39)	184.44 (156.51)	0.005	0.004	>0.1	>0.1
AFL surface [mm^2^]	16,436.85 (5201.20) *	18,427.35 (8913.10)	23,061.94 (12,065.20)	0.005	0.004	>0.1	>0.1
ILT volume [cm^3^]	261.38 (144.49)	200.30 (143.89)	197.16 (160.23)	0.009	0.013	0.051	>0.1
ILT surface [mm^2^]	41,692.89 (11,309.40)	41,143.71 (14,457.70)	44,225.26 (18,255.84) *	>0.1			
Calcifications volume [cm^3^]	3.01 (2.52)	2.31 (2.12)	1.54 (1.00)	<0.001	<0.001	>0.1	0.001
Calcifications surface [mm^2^]	2716.76 (2637.93)	2412.74 (2306.52)	1993.67 (859.57)	<0.001	<0.001	>0.1	<0.001
AFL/ILT volume ratio [cm^3^]	0.63 (0.50)	1.22 (1.03)	1.35 (0.78)	<0.001	<0.001	0.011	>0.1
AFL/ILT surface ratio [mm^2^]	0.39 (0.10)	0.45 (0.12)	8.07 (0.12)	<0.001	<0.001	0.033	0.016
Calcifications/ ILT volume ratio [cm^3^]	0.01 (0.01)	0.02 (0.02)	0.01 (0.01)	0.004	0.037	>0.1	0.005

* As indicated, for the values with normal distribution, the standard deviation is given in brackets. In other cases, interquartile range (IQR) is determined. *p*-value < 0.05 was considered statistically significant; aAAA—asymptomatic abdominal aortic aneurysm; sAAA—symptomatic abdominal aortic aneurysm; rAAA—ruptured abdominal aortic aneurysm; AFL—aneurysm flow lumen; ILT—intraluminal thrombus, IQR—interquartile range; SD—standard deviation.

**Table 3:** Univariate logistic regression analysis for the occurrence of aneurysm rupture compared to the group of asymptomatic aneurysms with their statistically significant cut-off values. * AUC values were not statistically significant and an analysis of cut-off values was not performed. AFL—aneurysm flow lumen, ILT—intraluminal thrombus.

Variables	OR	95% CI	*p*-Value *	Cut-Off Value	OR	95% CI	*p*
AFL volume [cm^3^]	1.010	1.004–1.016	<0.001	>181.56	7.170	2.428–21.174	<0.001
AFL surface [mm^2^]	1.000	1.000–1.000	0.002	>20,492.20	5.953	2.410–14.701	<0.001
ILT volume [cm^3^]	0.997	0.993–1.000	0.040	<172.16	4.786	1.957–11.704	<0.001
ILT surface [mm^2^]	1.000	1.000–1.000	>0.1	*	*	*	*
Calcifications surface [mm^2^]	1.000	1.000–1.000	>0.1	<782.12	8.366	3.294–21.249	<0.001
Calcifications volume [cm^3^]	0.861	0.722–1.027	>0.1	<1.35	8.044	3.218–20.105	<0.001
AFL/ILT volume ratio [cm^3^]	2.643	1.373–5.087	0.004	>0.43	11.316	4.042–31.360	<0.001
AFL/ILT surface*100 ratio [mm^2^]	1.187	1.099–1.281	<0.001	>0.40	13.895	4.676–41.285	<0.001
Calcifications/ILT volume*100 ratio [cm^3^]	0.838	0.641–1.096	>0.1	*	*	*	*

**Table 4:** Univariate logistic regression analysis for the occurrence of aneurysm rupture compared to the group of symptomatic aneurysms with their statistically significant cut-off values. * AUC values were not statistically significant and an analysis of cut-off values was not performed. AFL—aneurysm flow lumen, ILT—intraluminal thrombus.

Variables	OR	95% CI	*p*-Value	Cut-Off Value	OR	95% CI	*p*
AFL volume [cm^3^]	1.004	1.000–1.009	0.049	>189.26	2.591	1.077–6.232	0.034
AFL surface [mm^2^]	1.000	1.000–1.000	0.041	>18,808.70	3.474	1.502–8.033	0.004
ILT volume [cm^3^]	1.000	0.997–1.003	>0.1	*	*	*	*
ILT surface [mm^2^]	1.000	1.000–1.000	>0.1	*	*	*	*
Calcifications surface [mm^2^]	1.000	1.000–1.000	>0.1	<1090.98	7.153	2.874–17.801	<0.001
Calcifications volume [cm^3^]	0.915	0.777–1.077	>0.1	<1.57	6.250	2.353–16.598	<0.001
AFL/ILT volume*100 ratio [cm^3^]	1.001	0.998–1.004	>0.1	*	*	*	*
AFL/ILT surface*100 ratio [mm^2^]	1.045	1.004–1.087	0.03	>0.45	2.991	1.310–6.826	<0.001
Calcifications /ILT volume*100 ratio [cm^3^]	0.769	0.568–1.042	>0.1	<0.01	2.450	1.050–5.713	0.038

**Table 5:** Univariate logistic regression analysis for the asymptomatic aneurysms compared to the group of asymptomatic aneurysms with their statistically significant cut-off value. * AUC values were not statistically significant, and an analysis of cut-off values was not performed AFL—aneurysm flow lumen, ILT—intraluminal thrombus.

Variables	OR	95% CI	*p*-Value	Cut-Off Value	OR	95% CI	*p*
AFL volume [cm^3^]	1.005	1.000–1.011	>0.1	*	*	*	*
AFL surface [mm^2^]	1.000	1.000–1.000	>0.1	*	*	*	*
ILT volume [cm^3^]	0.996	0.993–1.000	0.027	<170.12	3.450	1.413–8.426	0.007
ILT surface [mm^2^]	1.000	1.000–1.000	>0.1	*	*	*	*
Calcifications surface [mm^2^]	1.000	1.000–1.000	>0.1	*	*	*	*
Calcifications volume [cm^3^]	0.919	0.794–1.065	>0.1	*	*	*	*
AFL/ILT volume*100 ratio [cm^3^]	1.006	1.001–1.011	0.022	>0.47	3.552	1.544–8.170	0.003
AFL/ILT surface*100 ratio [mm^2^]	1.067	1.017–1.119	0.008	>0.41	2.966	1.305–6.744	0.01
Calcifications/ILT volume*100 ratio [cm^3^]	1.060	0.873–1.287	>0.1	*	*	*	*

## Data Availability

The data are available on special request.
